# Three-dimensional mapping of microcircuit correlation structure

**DOI:** 10.3389/fncir.2013.00151

**Published:** 2013-10-10

**Authors:** R. James Cotton, Emmanouil Froudarakis, Patrick Storer, Peter Saggau, Andreas S. Tolias

**Affiliations:** ^1^Department of Neuroscience, Baylor College of MedicineHouston, TX, USA; ^2^Department of Bioengineering, Rice UniversityHouston, TX, USA; ^3^Department of Computational and Applied Mathematics, Rice UniversityHouston, TX, USA

**Keywords:** acousto-optical deflectors, two-photon imaging, motion-tracking, correlation structure, microcircuit activity, population coding

## Abstract

Great progress has been made toward understanding the properties of single neurons, yet the principles underlying interactions between neurons remain poorly understood. Given that connectivity in the neocortex is locally dense through both horizontal and vertical connections, it is of particular importance to characterize the activity structure of local populations of neurons arranged in three dimensions. However, techniques for simultaneously measuring microcircuit activity are lacking. We developed an *in vivo* 3D high-speed, random-access two-photon microscope that is capable of simultaneous 3D motion tracking. This allows imaging from hundreds of neurons at several hundred Hz, while monitoring tissue movement. Given that motion will induce common artifacts across the population, accurate motion tracking is absolutely necessary for studying population activity with random-access based imaging methods. We demonstrate the potential of this imaging technique by measuring the correlation structure of large populations of nearby neurons in the mouse visual cortex, and find that the microcircuit correlation structure is stimulus-dependent. Three-dimensional random access multiphoton imaging with concurrent motion tracking provides a novel, powerful method to characterize the microcircuit activity *in vivo*.

## Introduction

The outermost part of the brain—the cerebral cortex—is a 3D sheet of neural tissue that contains billions of nerve cells communicating through trillions of connections. Research over the last five decades has shown that single neurons in the cortex perform complex and subtle computations. However, little is known about how cortical neurons interact with each other to acquire their functional properties and how ensemble activity patterns are organized to process information.

Interestingly, anatomical connectivity between cortical neurons is locally dense via both vertical and horizontal connections (Gilbert and Kelly, 1975; Douglas and Martin, 2004; Song et al., 2005; Yoshimura et al., 2005; Thomson and Lamy, 2007; Otsuka and Kawaguchi, 2009; Yu et al., 2009). Moreover, the local wiring of the cortex is remarkably specific, demonstrating local higher order connectivity motifs (Song et al., 2005; Perin et al., 2011) and increased connection probability between more similarly tuned neurons (Ko et al., 2011, 2013). With the development of high throughput electron microscopy of brain tissue, the complete wiring diagrams of small volumes of neocortex may be available in a few years (Denk and Horstmann, 2004; Livet et al., 2007; Bock et al., 2011; Helmstaedter et al., 2013). Given the locally dense, specific, and three-dimensional nature of cortical connectivity, it is important to simultaneously monitor the activity of a large number of nearby neurons in 3D to link our understanding of anatomical connectivity (structure) with population activity (function) in order to unravel the underlying computations. For example, measuring the simultaneous activity of large populations of neurons *in vivo* is necessary for deciphering the principles of population coding. Theoretical work has shown that the precise structure of correlated neuronal variability, so called noise correlations, can significantly enhance or diminish the information capacity of neural ensembles (Zohary et al., 1994; Abbott and Dayan, 1999; Sompolinsky et al., 2001; Wilke and Eurich, 2002; Averbeck and Lee, 2004; Shamir and Sompolinsky, 2004; Ecker et al., 2011). However, these studies typically rely on extrapolating the population correlation structure from data recorded with pairs of neurons, but the assumptions behind this extrapolation can dramatically change the results (Shamir and Sompolinsky, 2006; Ecker et al., 2011; Pernice et al., 2013). Therefore, it is important to decode real populations to validate these theoretical studies and ultimately determine the impact of correlations on population coding (Graf et al., 2011; Berens et al., 2012). Simultaneously recording the activity of large population of neurons from local microcircuits has been hindered by the lack of appropriate methods.

Electrophysiology, while providing excellent temporal resolution, is limited in terms of scalability, recording density, utilizing fluorescent cell markers, and the precise localization of neurons. Alternatively, two-photon fluorescence laser-scanning microscopy (Denk et al., 1990) and population-staining methods (Stosiek et al., 2003) enable mapping the properties of all cells in a local volume with excellent spatial resolution. However, traditional *in vivo* two-photon microscopy works by moving mechanical components, which limits temporal resolution due to inertia. Moreover, scanning axially is even more inertia-limited due to the mass of the objective lens (e.g., 10 Hz) (Göbel et al., 2007). So while all the cells in a volume can be characterized independently, these methods are limited for studying their coordinated activity and interactions.

Three-dimensional two-photon imaging using acousto-optic deflectors (AODs) circumvents these limitations by allowing fast random-access 3D imaging (Reddy and Saggau, 2005; Reddy et al., 2008). Inertia-free scanning within a single plane has been applied successfully for *in vivo* studies using two acousto-optic deflectors (Grewe et al., 2010) and spatial light modulators (Nikolenko et al., 2008). Inertia free imaging also has the benefit of hopping directly between neurons, and thus not wasting time imaging from locations that are not of interest (i.e., neuropil). More recently, 3D-RAMP scanning with AODs was demonstrated *in vivo* to measure the orientation tuning preference of neurons (Katona et al., 2012). However, one major drawback of using random access scanning for *in vivo* research is that without an image sequence (Katona et al., 2012), it is challenging to determine if coordinated changes in fluorescence across the population are attributable to neural activity or movement. Because the main purpose of population recording is to characterize the coordinated ensemble activity, this is a critical limitation.

Here we describe the design of a microscope for *in vivo* studies using 3D random-access multi-photon (3D-RAMP) excitation that can perform neural population recording and simultaneous 3D motion tracking of the tissue by interleaving imaging from orthogonal planes. This was made possible with a custom field programmable gate array (FPGA)-based real time controller capable of continuously scanning complex patterns. The microscope employs four acousto-optic deflectors instead of traditional galvanometric and piezo scanning devices. Therefore, it can generate any desired 3D scanning path with a temporal resolution that is one to two orders of magnitude faster than current *in vivo* 3D two-photon imaging systems. We used this method to record the activity from populations of up to 411 cells in layers 2/3 of the primary visual cortex of the mouse and characterized the activity structure of local populations of neurons.

In agreement with previous reports (Kohn and Smith, 2005; Ecker et al., 2010; Denman and Contreras, 2013) we found higher noise correlations for pairs of neurons with similar tuning preference than more dissimilarly tuned pairs. In addition, we found a stimulus dependence of the correlation structure, where presentation of a stimulus near the preferred orientation of a pair of cells caused higher noise correlations than presentation of non-preferred stimuli.

## Materials and methods

### 3D-RAMP microscope

**Figure 1:**
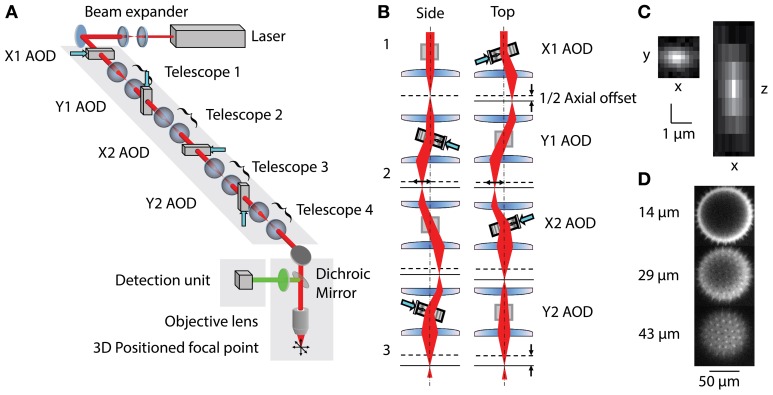
**(A)** Layout of 3D-RAMP Microscope. The expanded beam of a pulsed Ti:S laser is shaped in its collimation and angle at the backfocal plane of the objective lens by a chain of 4 AODs and telescopes, resulting in 3D positioned focus. **(B)** Principle of controlling the axial position of the laser focus. For clarity, a side and a top view of the scanning unit are shown. The direction and frequency of the chirped sound waves at two pairs of AODs creates the desired focal offset. Note that for simplicity a case with no desired lateral offset is shown here. This helps visualizing how the 2nd AOD of every pair compensates for the unwanted lateral displacement of the beam by the chirp. **(C)** Point spread function. Square figure to the left shows PSF in horizontal plane and figure to right shows axial section through PSF. **(D)** Spiny pollen imaged at 3 axial depths. Number to the left indicates offset from natural focal plane.

The 3D-RAMP scanner contains four AODs (OAD-1121, Isomet, VA) using TiO_2_ as the acousto-optical medium. The AODs are connected by 1:1 telescopes (200 mm focal length lenses) to optically map the pivot points of all deflectors into the back focal plane of the objective (Figure 1). The light source is a tunable ultrafast Ti:S laser (Chameleon Vision II, Coherent). The overall system efficiency (output/input power) is about 14%. The expanded laser beam diameter is 10 mm throughout, and imaging is performed with a water immersion objective (20x 1.0NA, Olympus, Japan) mounted to a modified open frame microscope (Moveable Objective Microscope, Sutter Instruments). The collection path is split by a dichroic mirror into the longer wavelength (red channel) and shorter wavelength (green channel) and measured with two high quantum efficiency GaAsP photomultiplier tubes (H7422P-MOD, Hamamatsu, Japan).

Three-dimensional imaging is performed using a pair of AODs for both the X and Y axis (Reddy et al., 2008). By driving a deflector with a linear frequency sweep (chirp), a beam with cylindrical phase is generated with a time-dependent mean angle (Figure 1B1). Pairing two deflectors, each with the sound propagating in opposite directions, the time-dependent component can be removed and the cylindrical phase curvature doubled. In this scheme, a difference in the frequencies creates a desired residual lateral offset. In order to achieve spherical phase curvature and lateral offset in the perpendicular direction, an orthogonal pair of deflectors was used (Figure 1B2). The same frequency ramp was used across all four AODs, creating the spherical curvature required for axial scanning and independent control of lateral positioning (Figure 1B3). This scheme allows moving the two-photon excitation point arbitrarily in three dimensions. To hop to a new location, the appropriate acoustic signal is presented to the deflectors, and the collected signal is blanked for the time it takes the new sound to propagate across the deflector crystal (10 μs). The dwell time was set to 10 μs, giving a time per point of 20 μs or an overall rate of 50,000 samples per second. This optical configuration results in a field-of-view of 200 × 200 μ m laterally and 100 μm axially. More details of the principles underlying 3D-RAMP microscopy have been described previously (Reddy and Saggau, 2005; Reddy et al., 2008) and a photograph of the system can be found in Supplementary Figure 1. In our system, a mirror on a motorized mount allowed remote switching of the illumination path between a traditional galvanometric scanning system and the 3D-RAMP scanner.

To ensure correct alignment and function of the deflectors, we measured the wavefront tilt and curvature entering the back-focal aperture of the objective lens using a wavefront sensor (WFS150-7AR, Thorlabs, Newton, NJ). We swept each individual AOD back and forth through its frequency range while holding the others at the center frequency. This confirmed that the wavefront tilt per MHz of frequency change was the same for each AOD, which would not be the case if the telescopes were misadjusted. In addition, this procedure verified that each AOD tilted only in either the X or Y axis and that there was no rotational misalignment. The tilting procedure was repeated with the beam diameter limited to 1 mm and imaged with a standard camera to verify that all AODs were optically pivoting in the backfocal plane, which verified the AOD placement along the optical path.

Another important alignment procedure was to repeatedly hop between the center frequency and a frequency with very low efficiency for each AOD while focusing in fluorescein solution, in order to time the latency from the hop time to the change in fluorescence. When each AOD was correctly centered along its acoustic axis the latency was the same and approximately equal to the propagation time across the AOD (10 μs with our AODs).

### Control module

To efficiently perform *in vivo* 3D-RAMP experiments, we needed a control system that could generate complex scan patterns continuously for long periods of time and simultaneously record voltage traces such as electrophysiology. We developed a new control system and matching user-interface software. Frequency ramps are generated using a four-channel digital synthesis board (AD9959 Evaluation Board, Analog Devices, Norwood, MA), which is updated synchronously across all channels. Two cascaded RF amplifiers (IA100 and DA104–2, Isomet) provide the 1.5 watts of RF power required to efficiently drive the AODs. The parameters for the subsequent ramp are clocked into the AD9959 during the hopping and dwell time of the previous point by custom firmware running on a field-programmable gate array (FPGA, PXIe-7965R with NI-5751 adapter module, National Instruments, Austin, TX) through a digital interface. The 16-channel, 14-bit, 50-MHz adapter module for the FPGA acquires the photomultiplier tube and electrophysiology data, which is then demultiplexed and pre-processed by the FPGA. Demultiplexing the optical signals involves blanking the data during the transition from one point to another and binning it during the dwell time. The FPGA then sorts the data into points from the functional scan and points from the motion planes (see Motion Tracking below). Voltage traces from the continuous channels (e.g., electrophysiology) are downsampled to the scanning rate (50 kHz). Running the code on an FPGA guarantees the difficult real time constraints required for this type of system are met.

**Figure 2:**
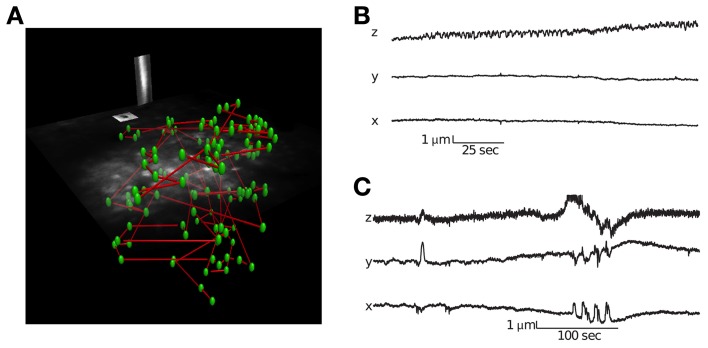
**Visualization of a scanning path and interleaved motion tracking planes. (A)** The location of all cells is labeled green and the scanning path is labeled blue. Interleaved into this scanning path are points located in two orthogonal motion tracking planes placed over a vessel and neuroglia. Motion tracking is computed from these orthogonal image planes in 3D (see Methods for details). **(B)** Movement traces from a session with little lateral movement but axial drift. **(C)** Movement traces from a session with large abrupt movements.

The corresponding user interface software allows one to perform a volume scan and select cells using a semi-supervised method that identifies the three dimensional center of neurons (see Supplementary Movie 1 for visualization of experimental paradigm). It is important to record signals from the center of the cell bodies in order to minimize neuropil contamination (Göbel and Helmchen, 2007). The user interface also allows placing motion-tracking planes in three dimensions over high contrast objects (Figure 2). During imaging, all electrical channels and the calcium fluorescence traces can be visualized in real-time.

### Surgical methods, dye loading, and electrophysiological recording

All procedures performed on mice were conducted in accordance with the ethical guidelines of the National Institutes of Health and were approved by the Baylor College of Medicine IACUC. Male C57CL/6J mice (age: p40–p60) were initially anesthetized with Isoflurane (3%) and anesthesia was maintained by either Isoflurane (2%) or a mixture of Fentanyl (0.05 mg/kg), Midazolam (5 mg/kg), and Medetomidin (0.5 mg/kg), with anesthesia boosts of half the initial dose administered every 3 h. The temperature of the mouse was maintained between 36.5 and 37.5°C throughout the whole procedure using a homeothermic blanket system (Harvard Instruments). In some experiments, the cardiac and respiratory rate were simultaneously monitored and recorded using a MouseOx (Starr Life Sciences Corp). Part of the skin over the skull was resected and the location of the craniotomy determined (approximately 2.5–2.9 mm lateral to the mid-line sagittal suture and anterior to the Lambda suture). A head-post bar was secured to the skull using bone cement, then a 0.5–1.5 mm craniotomy was performed over the region of interest. The mouse was transferred to a traditional galvanometric imaging system with a 10x objective (Nikon CFI Plan Apo 10×) for two-photon guided injection of the membrane-permeable calcium indicator Oregon green 488 BAPTA-1 AM (OGB-1, Invitrogen) and the astrocyte specific fluorescent dye sulforhodamine 101 (SR-101, Invitrogen). Dye solution was prepared as previously described (Garaschuk et al., 2006) and bolus loading injections were performed (Wu and Saggau, 1994; Stosiek et al., 2003). We used a pulsed low pressure protocol with a glass micropipette and a computer controlled pressure system (Picospritzer II) to inject ~300 μm below the surface of the cortex (Garaschuk et al., 2006). The window was then sealed using a glass coverslip secured with dental cement. After allowing 1 h for dye uptake, each injection resulted in a stained area of <400 μm in diameter.

To perform simultaneous juxtacellular recordings during two-photon calcium imaging recordings we replaced sulforhodamine 101 with Alexa Fluor 594 (Invitrogen) and used glass pipettes with 5–7 MΩ resistance for targeted two-photon guided recording as previously described (Komai et al., 2006).

### Motion tracking

**Figure 3:**
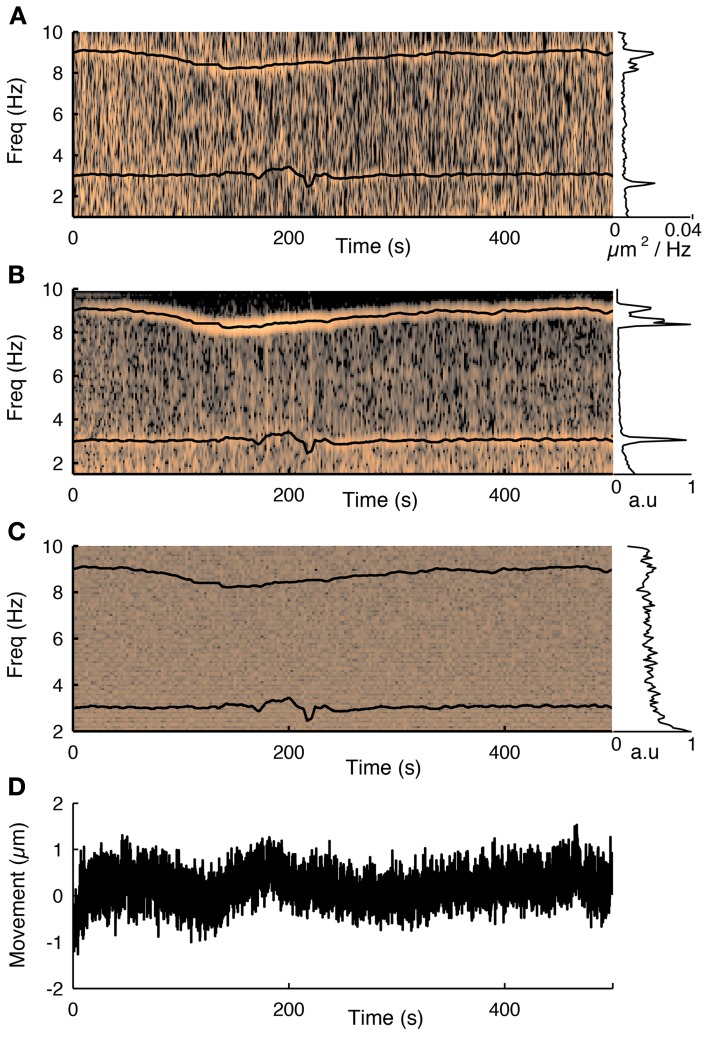
**The influence of the cardiac cycle on movement and calcium fluorescence. (A)** Spectrogram of the tissue movement computed from motion tracking planes (e.g., Figure 2A, see methods for details) shows a prominent narrow frequency band that changes over time between 7 and 10 Hz. The corresponding black trace is the heart rate measured simultaneously with a cardiac monitor. The frequency of the tissue movement matches the heart rate. A second frequency band around 3 Hz corresponds to the respiratory cycle, which was also monitored simultaneously and is shown with a black trace. The inset on the right shows the average spectrum over the course of the experiment and shows both these ranges having substantially more power than at other frequencies. **(B)** The same analysis performed on the first principal component of all fluorescence traces from 76 cells recorded during this session shows that the functional traces are also influenced by the cardiac and respiratory cycles. **(C)** Spectrogram of the average neuropil from the motion planes. The cardiovascular cycle does not affect the neuropil. **(D)** Estimated movement for this session. Despite the fact the movement is less than a micron it is sufficient to influence the traces.

To track the motion of the preparation during functional scanning, the 3D-RAMP scanner interleaves points on image planes with the functional calcium traces. This is performed by alternatively sampling a sequence of points on neurons and then a point from one of two motion-tracking planes, which are positioned over high contrast structures such as blood vessels or astrocytes (Figure 2). In order to track motion in 3D, two planes are used; one oriented parallel to the cortical surface and another perpendicular to it. The frame rate and resolution of these planes can be chosen based on the desired spatial and temporal accuracy. By interleaving the functional scanning coordinates with the motion tracking planes we acquire simultaneous functional data and structural movies (see Supplementary Movie 2). In practice we found imaging two 10 × 10 point planes spanning high contrast objects such as astrocytes or blood vessels at 10 Hz provided sufficient tracking accuracy without taking excessive imaging time from acquiring functional activity. These resolutions and speeds can resolve sub-micrometer movement while having enough bandwidth to detect both slow drifts and abrupt movements. Our custom user interface software allows visualizing the motion planes in real time during experiments. In the experiments where we wanted to compare the externally measured cardiac cycle to the motion estimate, we performed motion tracking at 25 Hz in order to adequately capture the cardiac cycle at 7–10 Hz (Figure 3).

Inferring a single motion estimate from these data is not straightforward for several reasons. Firstly, given that we record both a horizontal and vertical plane, which can share information about horizontal movement, these two planes must be combined together. Secondly, each plane collects two channels filtered at different wavelengths (called red and green below), each with different amounts of noise. Thirdly, because each plane contains only 100 pixels and each pixel is only imaged for 10 μs, the shot noise must be accounted for in order to optimally estimate position from all of this information. To solve this we developed a probabilistic motion-tracking algorithm that accounted for these issues. For each frame, the algorithm computes the three-dimensional offset of the tissue relative to the first frame.

We converted the recorded voltages into photon counts. The voltage measured by the photomultiplier tube is proportional to the photon rate, which for a fixed integration time of 10 μs is proportional to the photon count. For a given sampling location, the number of photons should be approximately a Poisson distribution with mean equal to variance (Wilt et al., 2013). The raw voltage was rescaled by a coefficient, which made the slope of the mean-variance plot for all the points of interest equal to unity. In practice, the photon rate is not perfectly constant over the course of an experiment, due to movement or neuropil fluorescence. We therefore computed the shot-noise variance as half the variance of the difference between sequential samples, which removes long timescale contributions from the total variance.

Movement was computed relative to a pair of orthogonal references, which define the mean photon count for each pixel in each plane and channel. These reference planes were initialized by averaging motion tracking movies over the first 10 s, then after the first iteration of the following motion-tracking algorithm, were recomputed with the whole stabilized sequence.

For each time step, we computed the likelihood as a function of 3D position offset. Because the shot noise for each image plane and channel is independent, the likelihood can be factorized into likelihood functions for each plane:

$$\begin{array}{l} p\left(s_{h}^{r},s_{h}^{g},\;s_{v}^{r},\;s_{v}^{g}|x,y,z;u\right)=p\left(s_{h}^{r}|x,y,z;u_{h}^{r}\right)p\left(s_{h}^{g}|x,y,z;u_{h}^{g}\right)\\ \;p\left(s_{v}^{r}|x,y,z;u_{v}^{r}\right)p\left(s_{v}^{g}|x,y,z;u_{v}^{g}\right) \end{array}$$

The variables *s* corresponds to the image planes from a time step and *u* is the reference image planes defining zero offset. The variables *x, y, z* correspond to three-dimensional offsets relative to the reference plane. Subscripts indicate either the horizontal (h) or vertical (v) plane whereas superscripts indicate red (r) or green (g) channels. The likelihood for each plane as a function of offset can be further factorized across the pixels in each image, because their shot noise is also independent:

$$p\left(s_{h}^{g}|x,y,z;u_{h}^{g}\right)=\prod_{i,j}\text{Poiss}\left[s_{h}^{g}\left(i,j\right);\;u_{h}^{g}(i-x,j-y)\right]$$

where (*i, j*) index into the sample and reference images (in this case the pixels of the green channel of the horizontal plane).

This computes the likelihood of seeing that photon count from a Poisson distribution with a mean rate given a particular offset from the reference plane. The likelihood was treated as independent under movement perpendicular to the plane (notice z does not show up on the right side above), but this constraint could be relaxed using a three-dimensional reference volume instead of plane.

Finally, the point estimate of position for that frame is computed as the expected value of position:

$$\;E\left[x,y,z\right]=\underset{x,y,z}{∭}x\cdot y\cdot z\cdot p\left(x,y,z|s;u\right)\;dx\;dy\;dz$$

p(x,y,z|s;u)=1/Zp (s|x,y,z;u)p(x,y,z;u)

where 1/*Z* is a normalization constant to make the probability function integrate to one and *p*(*x, y, z; u*) is treated as a constant prior, so can be ignored.

The algorithm was validated visually over many sessions by watching the movies and the motion inference (see for example Supplementary Movie 2). To determine whether to include a scan for subsequent analysis we low pass filtered the motion traces at 2 Hz and discarded any sessions with a movement range of more than 3.5 μm of movement laterally or 4.5 μm of movement axially. Out of 20 sites recorded for this study, nine met this stability criterion. (http://toliaslab.org/code-and-algorithms/aod-motion-tracking-algorithm/).

### Visual stimulus

Anesthetized mice were presented with full field drifting gratings with 90% contrast, luminance of 10 cd/m^2^, spatial frequency of 0.08 cycles/°, and temporal frequency of 2 cycles/s. Two sets of stimuli were presented to each imaging site: the first to map directional tuning and the second to characterize the correlation structure. Directional tuning was mapped using a pseudo-random sequence of drifting gratings presented at sixteen equally spaced directions of motions changing at 2 Hz for 3 min. The correlation structure was mapped by presenting a drifting grating with the same parameters as described above. In this case each direction of motion was presented for 1 s, followed by a gray screen for 1 s. The direction of the grating was randomly selected for each presentation as either upward or rightward drifting grating. Each stimulus condition was presented at least 180 times.

### Data collection and preprocessing

**Figure 4:**
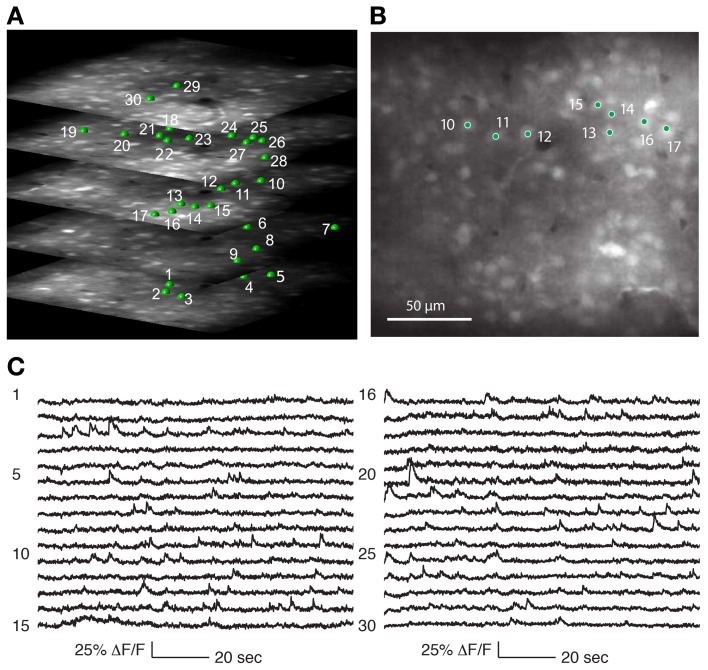
**Recording from a population of neurons with 3D-RAMP microscope. (A)** A 3D structural image is acquired by sequentially hopping through all the points in a 200 × 200 × 100 μm volume (250 × 250 × 50 points were acquired). Only a subset of the axial sections is shown. Cells are selected for functional calcium signals from each of the planes in the volume by clicking on the center of the cell bodies. In this data set 133 cells were recorded during spontaneous activity, of which 30 are shown here and are labeled green. **(B)** A plane from the middle of the stack, demonstrating that cells are clearly visible against the neuropil. **(C)** Functional calcium signals from 30 out of the 133 cells simultaneously recorded (each with 375 Hz sampling rate) are shown with the numbers corresponding to locations marked in **(A,B)**. Clear calcium transients are visible from the majority of cells. All signals were downsampled to 20 Hz for visualization. Signals from all of the 133 cells recorded are shown in Supplementary Figure 2.

**Figure 5:**
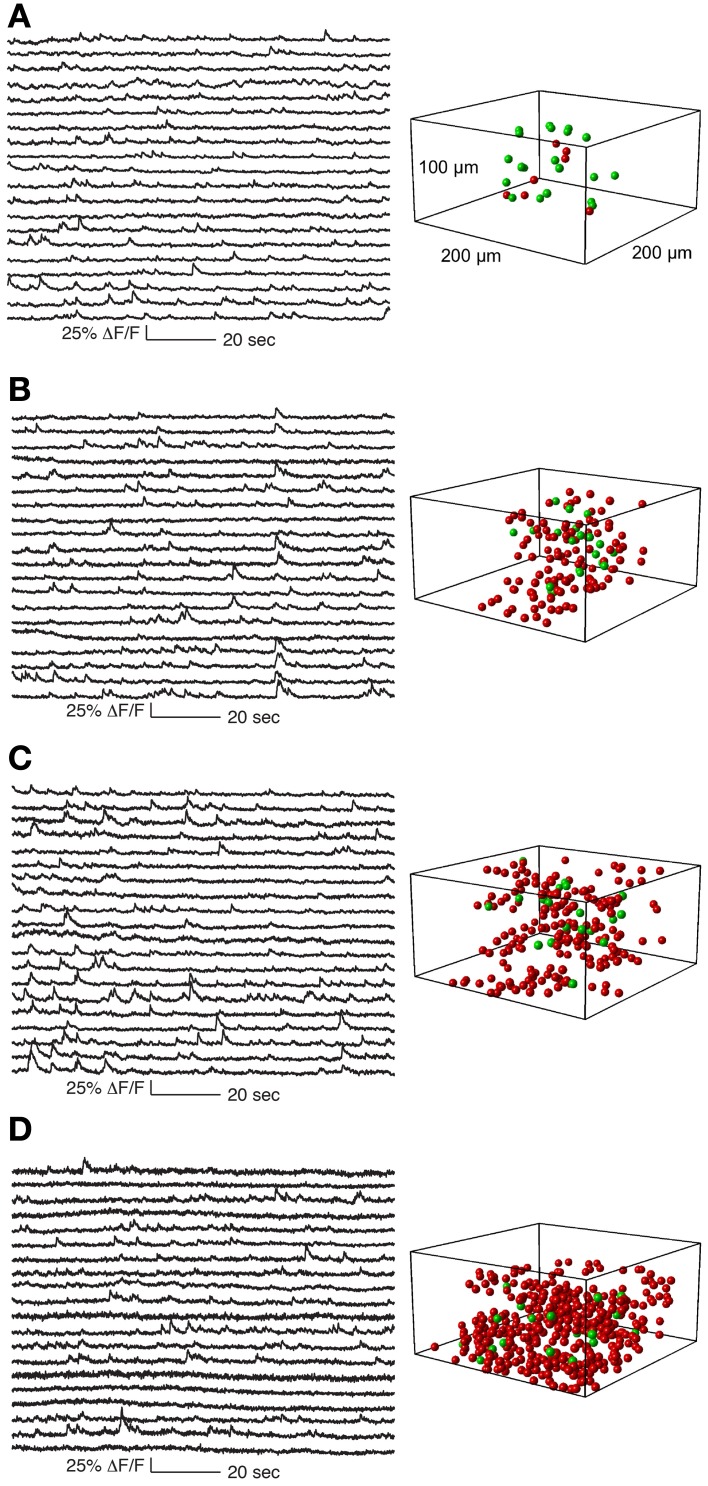
**Functional calcium signals recorded during spontaneous activity from data sets with different number of simultaneously recorded cells**. All data sets were collected from nearby sites. Locations of cells whose traces are shown on the left panel are labeled green on the right panel. Remaining cells recorded but whose traces are not shown are labeled red. The population sizes are: **(A)** 27 cells (at 1.8 Khz sampling for each cell), **(B)** 133 cells (375 Hz), **(C)** 205 cells (244 Hz), and **(D)** 411 cells (122 Hz). Functional traces are downsampled to 20 Hz for visualization and comparison. All traces from these datasets are shown in Supplementary Figures 2–5.

Three-dimensional structural volumes were acquired with the 3D-RAMP system. Cells of interest were selected by clicking on a cell, and then fitting a 5 μm radius sphere to the neuron to identify the 3D center. The recorded cells were between 100 and 300 μm below the pial surface. During random-access scanning the system repeatedly hops between the selected neurons to sample the calcium signals (Figures 4, 5).

The collected fluorescent traces were deconvolved to reconstruct the firing rates for each neuron. First, principal component analysis was performed on the population data, and the projections of the first principal component was removed from all traces (Bonin et al., 2011), which we found removed common mode noise related to small movement artifacts, and cardiovascular artifacts (Figure 3). The denoised result was bandpass filtered between 0.1 and 20 or 100 Hz. Inferred firing rates were estimated using a fast non-negative deconvolution algorithm (Vogelstein et al., 2010).

**Figure 6:**
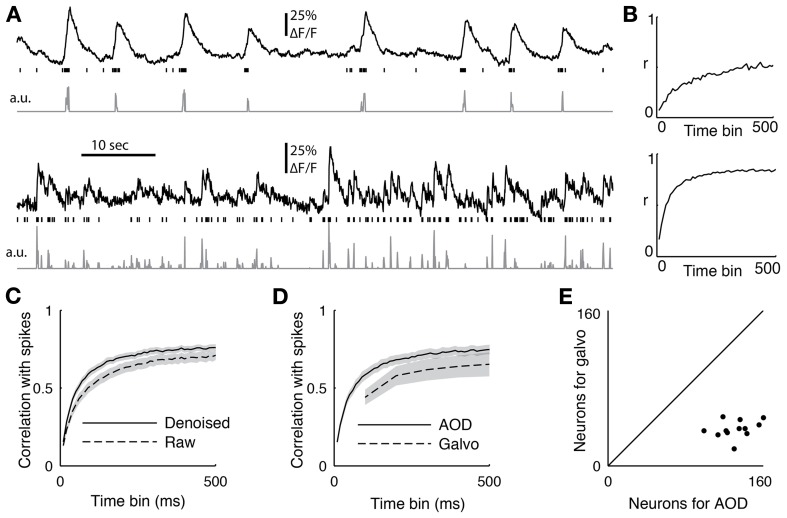
**(A)** Two sample cells recorded with functional imaging and electrophysiology. The top trace shows the downsampled fluorescence trace, the vertical lines show when spikes occurred, and the bottom trace is the deconvolved fluorescent trace. **(B)** The correlation coefficient between the spiking and the deconvolved trace binned at different time scales from 10 to 500 ms for the two sample cells **(C)** The average correlation coefficient between 18 cells with juxtacellular recordings and their firing rate inference comparing removing the first principal component of the activity (denoised) or not (raw). The PC removed data was significant better at all time bins (*p* < 0.05, ranksum test) **(D)** The average correlation coefficient between the spike count and the inferred firing from 12 cells recorded with both the AOD and galvanometric system. The error bar shows standard error of the mean, and the AODs recovered spiking significantly better than the galvos at 100 ms (*p* < 0.05, paired *t*-test). **(E)** The sizes of the populations recorded with both the galvanometric and 3D-RAMP system for data shown in panel d.

Spikes were extracted from juxtacellular recordings (see methods above) by bandpass filtering from 600 to 6000 Hz and detecting threshold-crossing events. The spike detection threshold was set manually for each cell and only cells with a high SNR were included. The quality of reconstruction was assessed by binning both spikes and deconvolved traces with time bins from 10 to 500 ms and computing the Pearson correlation coefficient (Figure 6).

### Data analysis

**Figure 7:**
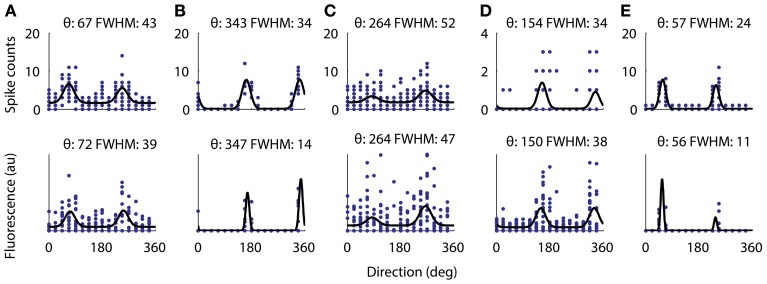
**Directional tuning curve recorded with electrophysiology from 5 cells (top) and the same tuning curves measured with functional imaging (bottom)**. All five cells were significantly tuned for a cosine fit as described in the methods. For each cell the fitted preferred orientation and tuning width are written above (width measured as full-width half-max). The largest difference between the fitted direction preferences as measured with electrophysiology from the measurements with AOD imaging was 5°. However, the two cells with very narrow tuning widths **(B,E)** have their width underestimated by the calcium imaging.

For computing tuning and noise correlations, data were low pass filtered at 20 Hz before deconvolving as we did not need higher temporal precision. Significant orientation tuning was determined by fitting a cosine function $r\left(\theta ;\theta_{p}\right)=A\cdot \cos\left(\frac{2\pi \left(\theta -\theta_{p}\right)}{180}\right)\;$ to the responses when presenting 16 directions of motion. A cosine function was fitted instead of a von Mises one because the subsequent analysis only depended on preferred orientation, and fitting additional parameters in a model reduces statistical power (however in Figure 7 where we compare tuning widths we fitted von Mises functions). A significant model fit was assessed by bootstrap analysis: first surrogate datasets were generated by randomly resampling responses 10,000 times with replacement while preserving the original presented orientation labels to create artificial datasets. The same cosine fit was performed for each surrogate dataset, and a cell was significant if the model fit to the real data explained more variance than model fits to 95% of the shuffled data sets.

The noise correlation matrix was computed separately for presentation of horizontal and vertical drifting gratings. For each trial the response for a neuron was defined as the deconvolved response averaged during the 1-s stimulus presentation. The correlation coefficient between two neurons for either horizontal or vertical stimulus trials was computed as the Pearson's correlation coefficient between their responses, (Figure 9).

## Results

### 3D imaging

The microscope was tested by measuring both point spread functions and visualizing pollen grains. Point spread functions were measured by imaging 100 nm yellow-green fluorescent beads (F8803, Invitrogen, CA) across the field-of-view and fitting a Gaussian to them, demonstrating a full-width half-maximum (FWHM) resolution of 0.5 μm laterally and 3 μm axially (Figure 1C). The 3D-RAMP scanner was used to image the fine 3D structure of 100 μm fluorescent pollen grains, clearly resolving ~1 μm-sized spines (Figure 1D).

We determined that the size of the 3D field-of-view that could be imaged simultaneously *in vivo* while retaining good signal quality was 200 × 200 μm laterally and 100 μm axially. Using Oregon Green BAPTA-1 as fluorescent calcium indicator, we obtained structural volumes by sequentially hopping between points on a 3D grid within this volume. Horizontal sections showed labeled neurons that were clearly distinguishable from neuropil at all axial positions (Figure 4A). We chose the grid spacing to trade off density of points against scanning speed, and typically used a spacing of 0.8 μm laterally and 1.5 μm axially. This allowed reliable identification of neurons and estimating the location of their centers (Figure 5).

### Motion tracking

Identifying movement during scans and only analyzing stable data is critical for characterizing population activity. We used a novel motion-tracking algorithm (see methods for details) to estimate movement from the pair of movies from motion planes (Figure 2A) and discard sessions with too much movement (see methods for details).

Using this approach, we were able to distinguish stable recording periods from those with significant tissue movement. Some sessions showed a steady drift that could also be identified by comparing the volume image before and after collecting functional traces (Figure 2B). However, other sessions exhibited abrupt movements, with no net drift (Figure 2C). In these cases, the simultaneous motion tracking was important in order to decide whether to keep the data for analysis. The position of each frame is computed independently, so the high frequency noise present in the traces in Figure 2 provides an indication of the amount of noise in the inference, which is well below 1 μm.

Next we showed that the cardiac and respiratory cycles induced movement that changed the measured fluorescence from the neurons. We performed simultaneous heart rate monitoring while imaging neural activity and tracking motion with our 3D-RAMP system. The spectrogram of the tissue movement showed a frequency band that tracked the heart rate remarkably well (Figure 3A) and the same frequency band was also present in the first principal component of the population activity (Figure 3B). We also monitored the respiratory rate of the animal and found that this contributed to a frequency band (~3 Hz) in both the motion estimate and the first principal component of the population activity (Figure 3). Despite the fact that the movement from the heartbeat and respiratory cycle had an influence on the functional traces, it was very small in magnitude, having only a root-mean-squared amplitude of 0.35 μm and range of about a micron (Figure 3C). To exclude the possibility the motion estimate came about from neuropil contamination or non-specific changes in fluorescence due to tissue oxygenation we computed the spectogram of the mean intensity of the motion tracking planes and did not observe any frequency bands (Figure 3D).

### *In vivo* functional imaging

We acquired functional data by sequentially hopping between neurons and sampling their calcium fluorescence. It has been shown that imaging neurons close to their center is important for reducing the amount of neuropil contamination (Göbel and Helmchen, 2007). To optimize this, coordinates were selected through a computer-assisted process that refined selected locations to the 3D center of neurons.

Calcium events were detectable from neurons throughout the 3D volume (Figures 4C, 5). As with any imaging technique, there is a tradeoff between the number of cells and the number of photons per cell, where more photons per second from a cell translates to a higher signal-to-noise ratio. With 3D-RAMP, the excitation volume sequentially “hops” between neurons, so the amount of time collecting photons from each cell is inversely proportional to the number of cells recorded. Time spent recording from motion-tracking planes also reduces the time available to collect photons from neurons.

In Figure 5 we show a typical experiment demonstrating how signal quality varied with the number of cells imaged. In this case we imaged four population sizes from the same recording site: 27 neurons sampled at 1.8 kHz, 133 at 375 Hz, 205 at 244 Hz and 411 at 121 Hz, (Figure 5). Clear calcium transients were visible from the majority of cells at all population sizes, although by 400 cells the amount of baseline noise begins to visibly increase. To illustrate the quality of our data, we show the traces of all cells recorded in these example four data sets (776 traces in total, Supplementary Figures 2–5).

### Firing rate inference

We performed simultaneous functional imaging and juxtacellular recordings from 18 neurons to quantify how well we could detect spiking activity from the functional imaging data. Figure 6A shows two examples of single-cell fluorescence signals, their recorded spiking activity, and the inferred firing rates computed using a fast non-negative deconvolution algorithm (Vogelstein et al., 2010). Bursts of spikes reliably caused the typical rapid rise in fluorescence followed by an exponential decay known as a calcium event. Although single spikes usually caused the same calcium events, they could not be reliably detected in all cells.

To quantify the quality of reconstruction at various time scales, we computed the correlation coefficient between the measured spikes and the inferred firing rates with bin sizes ranging from 10 to 500 ms (Figure 6C). When the spikes were binned at 50 ms, the average correlation coefficient across all 18 neurons between the spike counts and inferred firing rates was 0.48. This value increased to an average of 0.72 for a bin size of 250 ms and saturated by 500 ms to an average correlation coefficient of 0.76 (Figure 6C), which is the duration used here for measuring both orientation tuning and noise correlations for subsequent analysis.

We found that removing the first principal component of the activity recorded with AODs improved the reconstruction accuracy at all time bins from 10 to 500 ms (Figure 6C, *p* < 0.05, pairwise ranksum test). Importantly, the component that was removed correlated remarkably well with tissue movement (Figure 3) and increased the fraction of neurons that were significantly tuned to orientation (32% with PC removal vs. 22% without, *N* = 1451).

Next we compared the accuracy of reconstructing firing rates from data collected with the 3D-RAMP system and with data from a galvanometric scanner. We imaged with both the 3D-RAMP and a galvanometric system while recording juxtacellularly from the same neurons. We found that firing rates could be inferred more accurately with the 3D-RAMP system compared to the galvanometric system. Specifically, the correlation between the inferred firing rates at 100 ms bins and the measured firing rates was significantly higher for the 3D-RAMP system than the galvanometric system (Figure 6D, 3D-RAMP mean: 0.59, galvo mean: 0.44, *p* < 0.05, paired *t*-test, *N* = 18 cells). Importantly, the 3D-RAMP also has the advantage that it recorded on average three and a half times more cells than the galvanometric scanner (Figure 6E). Moreover, the reconstruction accuracy for the 3D-RAMP system was higher than published values using a resonance scanner system (3D-RAMP mean correlation 0.69, *N* = 18; resonance scanner mean correlation 0.53, *N* = 8 (Bonin et al., 2011); bin size 240 ms for both systems). In the case of the data we acquired with a galvanometric scanner (at 10 Hz), removing the principal component did not improve the reconstruction accuracy and thus was not performed (Figure 6). This difference is probably because at the high sampling rates of the AOD system the principal component analysis captures common noise artifacts, but at the lower frequencies of a galvanometric scanner it is dominated by true functional activity.

### Orientation tuning

**Figure 8:**
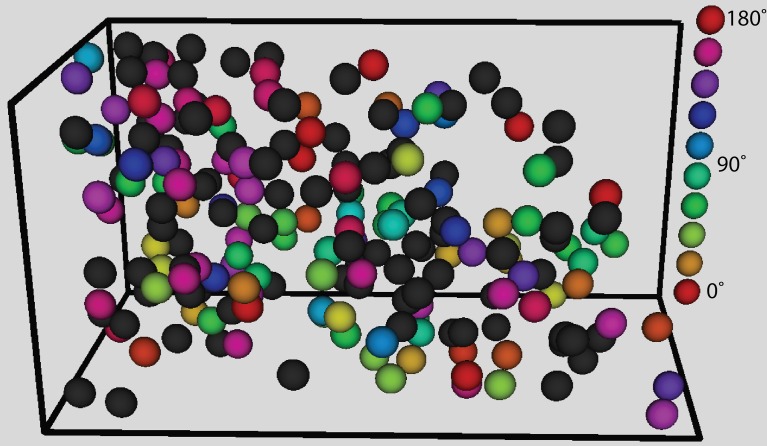
**The three-dimensional visualization of orientation preference for a single recording site**. Black spheres indicate cells that were not significantly tuned. The color of the other spheres indicates the orientation preference. The column on the right indicates the corresponding orientation indicated by the colors.

Next we verified that we could reliably and accurately measure the same tuning curve for V1 neurons with functional imaging as with electrophysiology. We recorded juxtacellularly from five cells in V1 while presenting oriented drifting gratings and computed their tuning curves from the inferred firing rate (Figure 7). For all five cells the preferred orientation was recovered accurately to within 5°. For three of the five cells the tuning width was recovered within 5° but for the two with the narrowest tuning the width it was underestimated by 13 and 15°, respectively (Figures 7B,E). This effect is most likely due to the non-linearities of two-photon imaging and spike inference suppressing weaker responses more than stronger ones. Overall, we found that 32% of 1454 neurons (9 sites, 6 mice, example site shown in Figure 8) were significantly tuned to stimulus orientation (see methods for details).

### Correlation structure

We next estimated the noise correlation structure. We only analyzed recording sites that met the stability criterion described in the Methods section (motion-tracking subsection). After mapping the directional tuning of populations of neurons in V1, we randomly interleaved gratings moving either upwards or rightwards to measure the noise correlation structure under these two stimulus conditions. Each stimulus was repeated at least 180 times. An important advantage of our technique is that we can estimate the full correlation matrix of a local population of neurons in contrast to electrophysiological methods, which can measure only a small sparse subset of its entries.

**Figure 9:**
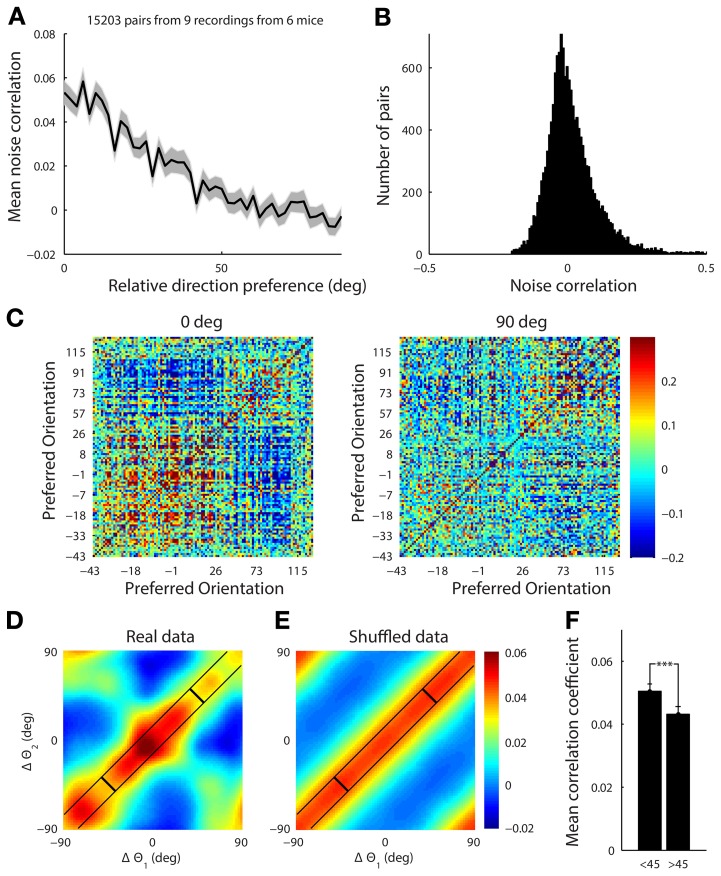
**(A)** The mean noise correlation between pairs of tuned neurons vs. their difference in orientation, with error bar representing the standard error of the mean. This shows a limited range structure **(B)** Histogram of the noise correlation value for all pairs of neurons. **(C)** The correlation structure from a single site with a simultaneously recorded population of 108 tuned neurons in response to presentation of horizontal (0°) and vertical (90°) gratings drifting upwards and rightwards, respectively. The neurons are ordered by their preferred orientation, which are indicated on the axes. The white line indicates the presented stimulus. It is clear that there is a higher correlation amongst neurons when the stimulus is near both of their preferred orientations. **(D)** This effect was characterized by collapsing across 9 datasets. For each stimulus the noise correlation, *r*, was measured for a pair of cells, and the difference between the stimulus (*s*, either horizontal or vertical) and the preferred orientation (θ) for each cell (Δθ = *s* − θ) was computed. The set of points from all the pairs of cells under both stimuli, (Δθ_1_, Δθ_2_, *r*), were then smoothed using a Gaussian kernel (Hastie et al., 2009) with a standard deviation of 10°. There is structure in both diagonal directions reflecting both limited range correlations and stimulus dependent changes in the correlation structure. **(E)** The presented stimulus orientation was shuffled to demonstrate what a purely limited range correlation structure with no stimulus dependence would look like. **(F)** Average noise correlation from all datasets for pairs of neurons with similar preferred orientation (within 10°) when the stimulus was near their mean preferred orientation (<45° column) vs. further away (>45°). ^***^p < 1e-4.

We found that neurons in the mouse visual cortex show a “limited-range” correlation structure (15,000 pairs of neurons, Figure 9A), where more similarly tuned neurons are more correlated—in agreement with previous electrophysiological studies in non-human primates (Kohn and Smith, 2005; Ecker et al., 2010) and mice (Denman and Contreras, 2013). For neurons tuned within 10° of each other, the median noise correlation was 0.019 (mean 0.040) compared to neurons tuned between 80 and 100° apart which had a median of −0.01 (mean 0.004, *p* < 0.05, ranksum test). The distribution of noise-correlations has a long positive tail although it contains a small fraction of the neurons, which indicates that despite the fact the noise correlations on average are low, some pairs are strongly coupled (Figure 9B).

We found that the noise correlation structure was also stimulus dependent. When horizontal gratings were presented, the set of cells preferring horizontal gratings had higher noise correlations than when vertical gratings were shown. The reverse was true when vertical gratings were presented (Figure 9C for single example site). To characterize the stimulus dependence of the correlation structure for all of our data, we computed the average noise correlation as a function of the difference between the stimulus and the preferred orientation of significantly tuned pairs of neurons (15,000 pairs of cells, 9 recording sites, 6 mice, Figure 9D). For each stimulus the noise correlation, *r*, was measured for a pair of cells, and the difference between the stimulus (*s*, either horizontal or vertical) and the preferred orientation (θ) for each cell (Δθ = *s* − θ) was computed. The set of points from all the pairs of cells under both stimuli, (Δθ_1_, Δθ_2_, *r*), were then smoothed using a Gaussian kernel (Hastie et al., 2009) with a standard deviation of 10°.

To show what the data would look like with no stimulus-dependence but preserving the limited-range structure, we repeated the analysis described above. However, for each pair of neurons we replaced the stimulus value, *s*, with a random stimulus orientation from 0 to 180 (Figure 9E). The surrogate dataset shows a diagonal-constant structure, where the noise correlation is constant along one diagonal direction and not the other. This is clearly different than the real structure shown in Figure 9D, which changes in both diagonal directions.

In order to quantify the stimulus dependence amongst pairs of neurons with a similar preferred orientation (within 10°), we measured the noise correlation when the difference between the stimulus and the average preferred orientation of the cells was less than 45° (mean = 0.051, Figure 9F) and when the difference was greater than 45° (mean = 0.043, Figure 9F) There was a significant difference between these two cases with stimuli near the preferred orientation producing higher noise correlations for the same pairs of neurons (Figure 9F, ranksum test, *p* < 1e-4).

## Discussion

We developed a 3D-RAMP microscope that can acquire functional calcium traces from large populations of neurons *in vivo* arbitrarily distributed in a 3D volume with excellent signal quality and high temporal resolution. The ability to rapidly focus on arbitrary locations in 3D makes it possible to apply two-photon imaging techniques to study the interactions amongst neurons spanning different cortical depths *in vivo*. This method enables one to record from hundreds of nearby neurons distributed in 3D (e.g., >400 cells within a 200 × 200 × 100 μm volume at a sampling rate of 120 Hz).

Simultaneous motion tracking is a challenge for any discontinuous hopping-based scanning technique that does not produce a structural image. Importantly, motion artifacts will contaminate the measured signals and cannot be straightforwardly distinguished from functional calcium fluorescence. Because one of the main motivations of 3D-RAMP scanning is to study large-scale population activity, such artifacts can significantly alter the results and in particular the correlation structure. Our approach enables, for the first time, simultaneous 3D tissue motion tracking by interleaving movies over high contrast structures with random-access functional scanning (3D-RAMP). This is absolutely necessary for any study of the structure of population activity using 3D-RAMP. Our implementation allows aborting scans with too much movement online or rejecting them *post-hoc*.

The imaging locations required for motion tracking consume time that could be otherwise used to collect functional traces, which will reduce the effective signal-to-noise ratio. For studying the tuning properties of neurons, simultaneous motion tracking is less necessary because movement that is uncorrelated with the stimulus can average out. However, tracking is an absolutely necessary cost for studying correlated activity *in vivo* because of the susceptibility of these measures to non-stationary (Brody, 1998; Bair et al., 2001; Ecker et al., 2010) such as movement. Comparing the position of the volume between the start and end of the experiment is not sufficient as brief and large motion transients can and do occur in the preparation (e.g., Figure 2C). The fraction of time spent collecting motion-tracking data is determined by the number, resolution, and sampling rate of the motion planes. We found two planes of 10 × 10 points at 10 Hz provided precise motion tracking while only consuming 15% of the imaging time.

An advantage of the FPGA scanner we implemented is that it can be extended to calculate the motion of the preparation in real-time and provide correction signals. Slower movements can be corrected by controlling the objective manipulator in 3D, and faster movements can be corrected within the FPGA itself by shifting the coordinates of all the points by the amount of movement measured.

We found that the inferred firing rates from the fluorescent activity correlated well with simultaneously recorded spike counts and our 3D-RAMP scanner outperformed traditional galvanometric scanner imaging the same cells, even while recording from a larger population (Figure 6). The 3D-RAMP scanner also outperformed published values for a resonance scanning system (Bonin et al., 2011). In this work, we did not optimize the parameters of the spike inference algorithm, as this requires many more neurons with simultaneous electrophysiology to robustly cross-validate the reconstruction accuracy. Accordingly, our current results should be taken as a lower bound on the performance of our 3D-RAMP system.

The method of firing rate inference used in this work (Vogelstein et al., 2010) does not estimate individual spike times and thus does not allow determining when false positives and false negatives occur. Extending this work to spike train estimation will be an important advance that will enable quantifying these types of errors and comparing the detectability of single isolated action potentials vs. bursts of spikes. When developing such algorithms it is critical to have large enough datasets to both train and cross-validate the algorithm in order to determine the error rates on novel data. In addition, a validated method with the ability to separate the cells that can be reconstructed accurately from those that cannot be would be a critical advance in the field.

Recently Katona et al. (2012) have demonstrated 3D-RAMP imaging from the visual cortex of the mouse. There are a number of similarities and differences between our system and theirs. The optical principles behind both systems are essentially identical to those described previously (Reddy and Saggau, 2005): four AODs are used in combination with the first pair providing axial focusing and the second pair providing additional axial focusing and lateral positioning. One difference is that the system described here places a telescope between each AOD in order to align the pivot point to the backfocal aperture of the objective. The Katona system used highly optimized AODs that allowed for a larger field of view compared to our system. It is challenging to compare the signal quality between the two systems because they did not report simultaneous *in vivo* functional imaging and electrophysiological recordings. The critical advance of our system over previous random-access imaging implementations is the simultaneous motion tracking as well as the quantification of signal quality, both of which are absolutely necessary for studying the larger scale properties of population activity. In addition our control system allows continuous recording from complex scan patterns.

We used the 3D-RAMP system to analyze the 3D microcircuit correlation structure. Measuring noise correlations is challenging. For example, non-stationarity or poor signal quality can significantly bias their estimate (Brody, 1998; Bair et al., 2001; Ecker et al., 2010). Measuring them with two-photon presents a host of additional challenges such as: (1) noise in the fluorescence signal can reduce the measured values, (2) artifacts such as neuropil contamination, anesthesia related activity, or movement can increase them, (3) the spike to fluorescence transformation is non-linear. For these reasons the absolute value measured with two-photon should not be directly compared to electrophysiology. For example, while removing the first principal component from the fluorescence data improves reconstruction accuracy and increases the fraction of neurons that are significantly tuned to orientation, it also reduces the average correlation value. While most of this reduction reflects removing noise artifacts from the recording method, such as submicron movement from the cardiac cycle, some of it could reflect correlated network activity. Despite these caveats, the ability to record the activity from hundreds of adjacent neurons makes two-photon imaging and in particular 3D-RAMP a powerful method for analyzing the microcircuit activity structure.

We used our method to map the noise correlation structure with dense coverage of the neurons within a small microcircuit. To our knowledge, this is the first time the noise correlation structure of such a large and dense microcircuit has been measured *in vivo*. The noise correlation exhibited a limited range structure, with more similarly tuned neurons being more correlated. This is in agreement with electrophysiological measurements from macaque visual cortex (Kohn and Smith, 2005; Ecker et al., 2010) and mice (Denman and Contreras, 2013) and also consistent with connectivity mapping studies in mouse visual cortex (Ko et al., 2011, 2013).

In many studies of noise correlations in visual cortex, multiple orientations are presented but the noise correlation is averaged across stimulus conditions. These studies have also demonstrated the averaged noise correlation is higher for pairs of neurons with higher average firing rates (Kohn and Smith, 2005; Ecker et al., 2010). By presenting just two stimuli many times (at least 180 repetitions in each condition), we were better able to characterize the correlation structure in each stimulus condition and detect systematic changes. We found that in addition to a limited range correlation structure, the noise correlation changes in a stimulus dependent manner. Specifically, pairs of similarly tuned neurons are more correlated when presented with a preferred stimulus compared to when presented a non-preferred stimulus. Josić et al. (2009) has shown this kind of change in the correlation structure can, in certain conditions, increase the information capacity of a neural population. Our result is not consistent with a correlation structure that only depends on the geometric mean firing rate averaged across conditions, but might be explained by the correlation depending on the firing rate under each stimulus condition (Smith and Kohn, 2008). It is challenging to test for this explicitly with two-photon imaging, because across neurons the size of the single action potentials evoked calcium event is variable. This introduces an arbitrary scale to the inferred firing rate.

Our current understanding of the impact of noise correlations on coding accuracy often relies on extrapolating from pairwise measurements to the larger populations (Zohary et al., 1994; Abbott and Dayan, 1999; Sompolinsky et al., 2001; Wilke and Eurich, 2002; Averbeck and Lee, 2004; Shamir and Sompolinsky, 2004). However, there is little to no empirical evidence to constrain or confirm these extrapolations, which can profoundly change the results (Shamir and Sompolinsky, 2006; Ecker et al., 2011). In order to directly test the role of correlations in neural coding it is imperative to decode from empirically measured populations (Berens et al., 2012). The 3D-RAMP method is ideally suited to studying information coding of large populations of nearby neurons.

The capabilities of *in vivo* 3D-RAMP microscopy that we demonstrate here open an exciting new dimension in the analysis of neural microcircuits. For example, we can record from many cells simultaneously in the vertical dimension with high temporal resolution in order to study information processing along microcolums in layers 2/3. The ability to selectively record from specific cells of interest arranged in 3D, also opens the window to study the computations single neurons within the microcircuit perform on their synaptic inputs (Wickersham et al., 2007; Marshel et al., 2010).

### Conflict of interest statement

The authors declare that the research was conducted in the absence of any commercial or financial relationships that could be construed as a potential conflict of interest.
