# Supplementary material for: Three-dimensional mapping of microcircuit correlation structure
Source: Front Neural Circuits. 2013 Oct 10;7:151. doi: 10.3389/fncir.2013.00151 (PMC3794294; doi:10.3389/fncir.2013.00151)
Supplement: Supplementary file 1 [file DataSheet1.PDF]

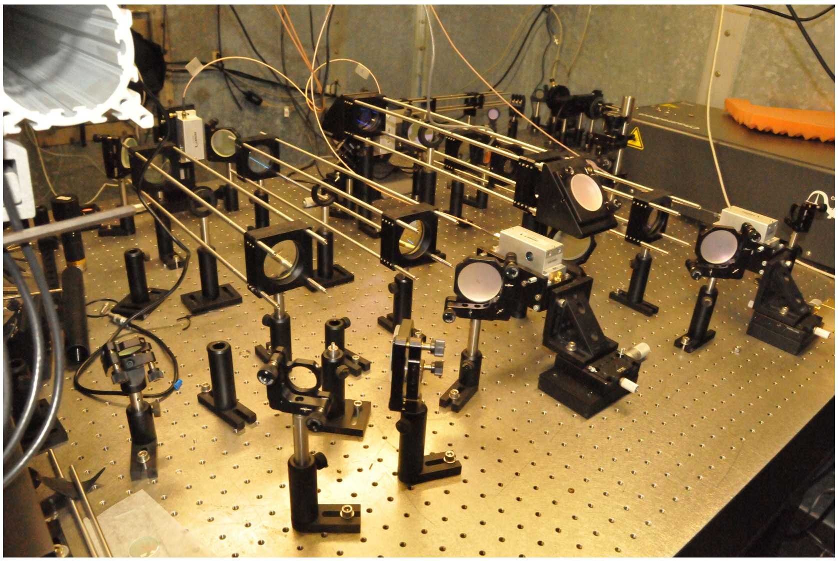

SUPPLEMENTARY FIGURE 1. This is a photograph of the AOD system described in this work. The silver boxes are the AODs, each mounted on a manipulator that allows changing the position along the optical path and rotating the Bragg angle. Between each AOD is a 1:1 telescope which maps all of the pivot points of the AODs into the backfocal aperture of the objective.

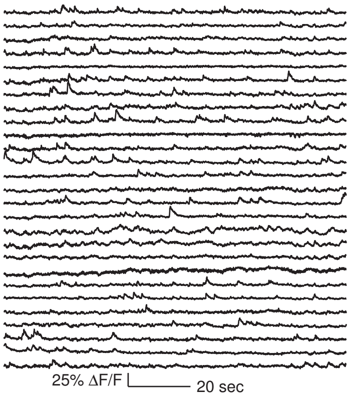

SUPPLEMENTARY FIGURE 2. Traces from all of the cells shown in Figure 5a (27 cells imaged at 1.8 kHz)

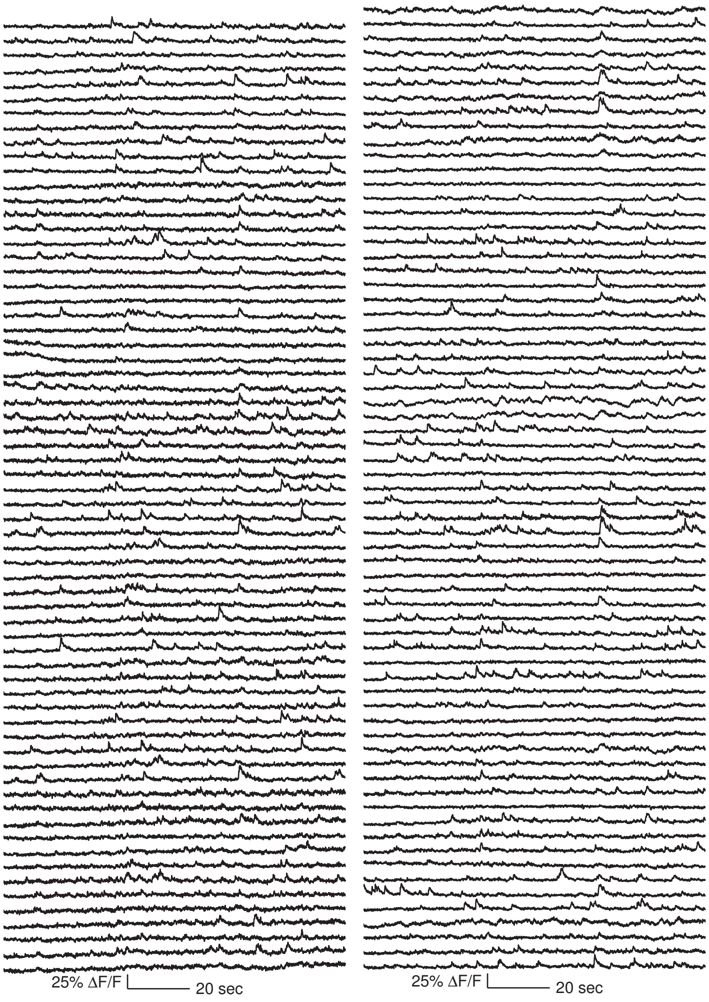

SUPPLEMENTARY FIGURE 3. Traces from all of the cells shown in Figure 5b (133 cells imaged at 375 Hz)

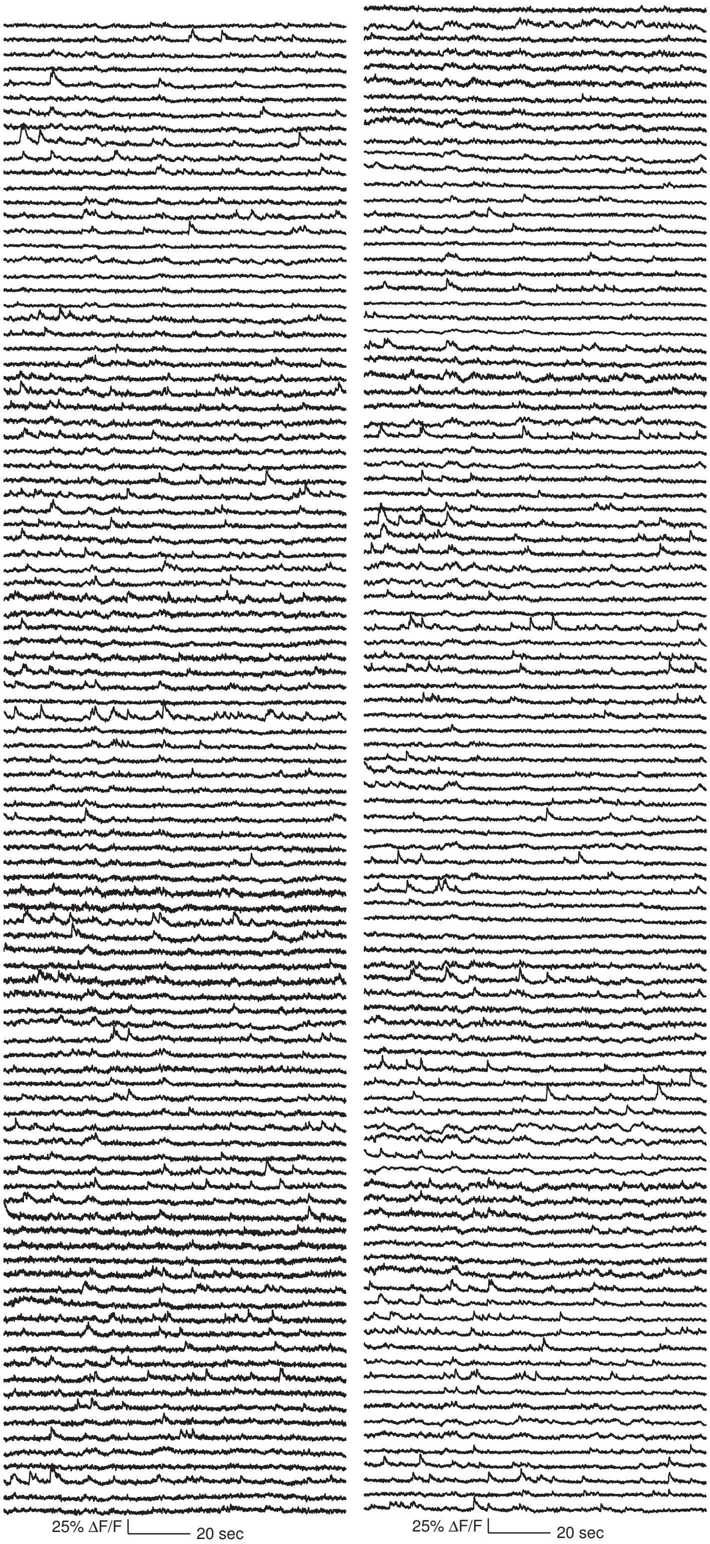

SUPPLEMENTARY FIGURE 4. Traces from all of the cells shown in Figure 5c (205 cells images at 244 Hz)

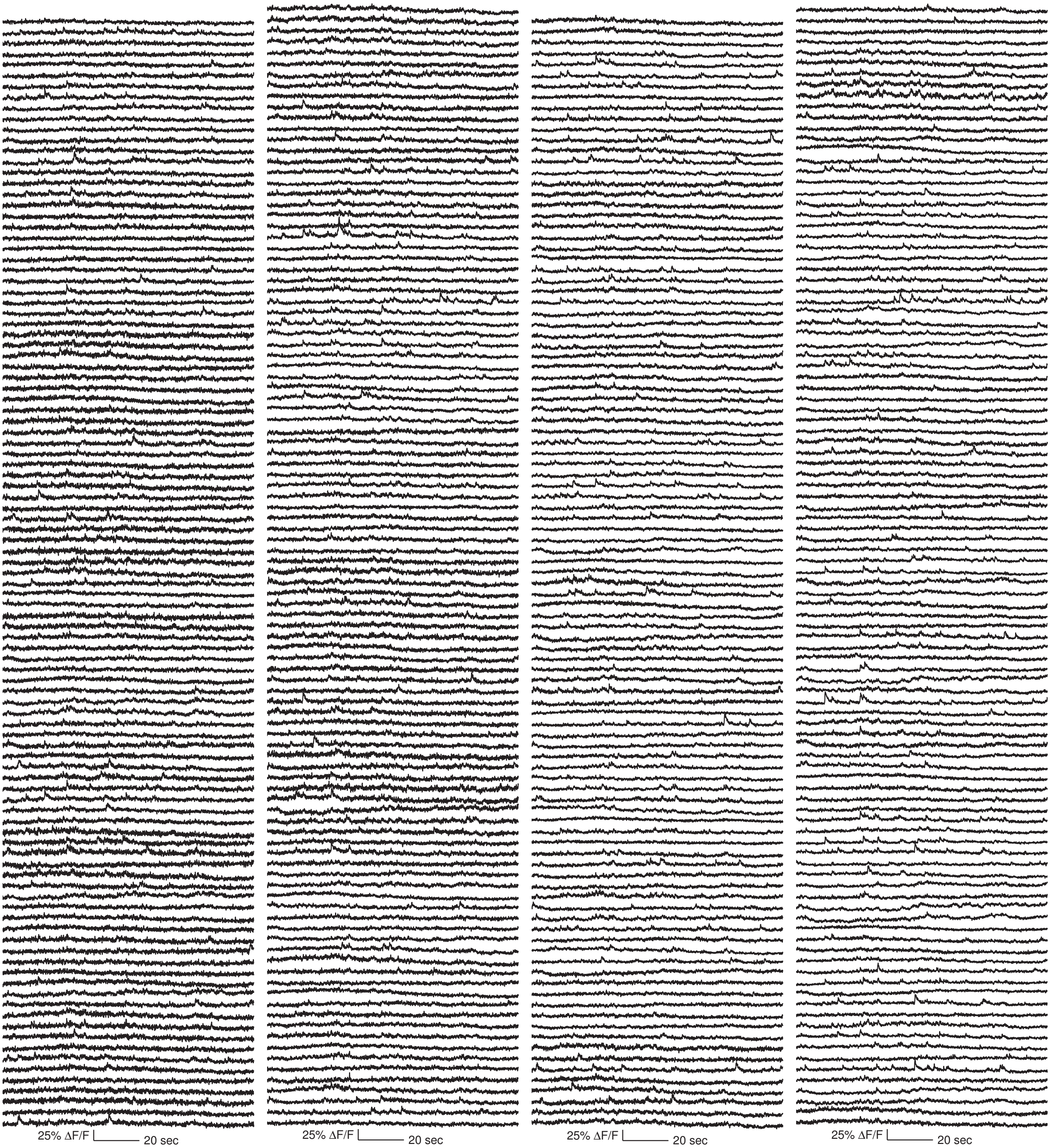

SUPPLEMENTARY FIGURE 5. Traces from all of the cells shown in Figure 5d (411 cells imaged at 122 Hz)
